# Effect of physically active learning on physical activity levels in secondary school students: The ACTIVE CLASS Study

**DOI:** 10.1186/s12889-026-26814-6

**Published:** 2026-03-07

**Authors:** María González-Pérez, Daniel Camiletti-Moirón, Abel Ruiz-Hermosa, Fátima Martín-Acosta, Alberto Grao-Cruces, David Sánchez-Oliva

**Affiliations:** 1 GALENO Research Group, Department of Physical Education, Faculty of Education Sciences University of Cadiz, Puerto Real, 11519 Spain; 2 Instituto de Investigación e Innovación Biomédica de Cádiz (INiBICA), Cadiz, Spain; 3 Social and Health Care Research Center, Universidad de Castilla-La Mancha, Cuenca, Spain; 4 Faculty of Education, Universidad de Castilla-La Mancha, Ciudad Real, Spain; 5 ACAFYDE research group, Department of Didactics of Musical, Plastic and Body Expression, Faculty of Sports Sciences, University of Extremadura, Caceres, Spain

**Keywords:** Active lesson, Adolescent, Middle school, Sedentary behaviour, Accelerometry

## Abstract

**Background:**

Currently, school days are predominantly sedentary, which has negative implications for students’ physical and mental health. This study had two primary objectives: (i) to evaluate the effects of a physically active learning (PAL) intervention on physical activity (PA) levels during school hours; and (ii) to compare PA patterns during school hours and throughout the day between days with and without PAL sessions. As a secondary objective, the study analysed the intensity distribution of PA during PAL lessons.

**Desing and Methods:**

This randomised controlled trial included 113 students (7th -8th grade) from four secondary schools. The experimental group (*n* = 60) received a weekly PAL lesson for 16-weeks, while the control group (*n* = 53) continued with traditional teaching. PA and sedentary time were assessed by accelerometers over seven full-days before and during the last week of the intervention. Generalised linear mixed models were used to analyse the intervention’s effect on school PA and the differences in full-day and school-time PA on PAL vs. non-PAL day. Additionally, a descriptive analysis was performed to present the average distribution of PA intensities during PAL lessons.

**Results:**

Although a slight reduction in sedentary time and an increase in moderate-vigorous PA (MVPA) were observed, no significant differences were found in PA levels during the overall school-time after the intervention, nor in full-day PA the day it was implemented. However, there was a significant effect on the day of implementation of a PAL lesson in MVPA levels (*p* < 0.001, η2 = 0.444) during school-time.

**Conclusions:**

PAL lessons can significantly increase MVPA during the school day, but these effects do not appear to persist throughout the full-day or in weekly school averages.

**Trial Registration:**

Clinical Trials Registry: NCT05891054. Retrospectively registered. https://clinicaltrials.gov/study/NCT05891054?cond=ACTIVE%20CLASS%20study&rank=1.

**Supplementary Information:**

The online version contains supplementary material available at 10.1186/s12889-026-26814-6.

## Background

The evolution of lifestyles in recent decades, characterised by increased screen time and reduced active travel, has created increasingly inactive and sedentary environments for children and adolescents [1]. Despite the well-known benefits of regular physical activity (PA) for physical and mental health, academic performance, and cognitive development [2–5], current data show alarmingly low PA levels among children, especially adolescents [6]. At a European level, fewer than 29% of children and adolescents comply with the World Health Organisation’s recommendation to accumulate at least 60 min of moderate-to-vigorous-PA (MVPA) daily [7, 8]. Furthermore, sedentary behaviours account for nearly half of their free time [9]. This trend is also evident in Spain, where only 36.2% of children and adolescents adhere to these guidelines [10].

As environments in which students spend most of their waking hours, schools have not escaped this trend and may even be reinforcing it. The American Heart Association recommends that children accumulate at least 30 min of MVPA during the school day [11]. However, current data show that fewer than a quarter of Spanish children and adolescents meet with this recommendation [12], devoting between 66% and 81% of their time at school to sedentary behaviour [13].

Although several social factors may contribute to this decline, the school environment could play a key role in finding a solution. Schools are structured and accessible environments that concentrate most children and young people for much of the day. This makes them ideal settings for implementing interventions that promote healthy lifestyles, including PA. Various initiatives have been developed for this purpose, mainly focusing on Physical Education, the transformation of school spaces (indoor and outdoor) to provide opportunities for PA, and the implementation of active recess strategies. However, these strategies appear to be not very effective in increasing daily PA levels, particularly among adolescents [14]. For this reason, an educational approach known as physically active learning (PAL) has gained increasing attention over the last decade. This strategy aims to integrate PA into core subjects that are traditionally taught in a sedentary way by incorporating movement-based learning tasks and dynamics [15]. In this sense, PAL can be understood as an approach that provides structured opportunities for movement within the classroom, aiming to reduce sedentary time without extending the school day or compromising academic instruction. It is important to acknowledge that the specific implementation of PAL (e.g., lesson frequency, duration, subject area, or activity intensity) may vary across studies. However, in all cases, by modifying the learning environment itself, PAL seeks to facilitate more active behaviours in a context where prolonged sitting is the norm.

Previous studies have analysed the impact of PAL on students’ PA levels. In primary education, evidence suggests that PAL could have a positive effect on PA, with benefits observed across several indicators. These include increases in overall PA [16], and in the number of daily steps [17]. Similar effects have been reported during the school day, where PAL implementation has been associated with reductions in sedentary time [18], and significant increases in PA [19], including MVPA [20] and the number of steps taken [21]. From a more specific perspective, studies focusing on PAL sessions themselves have shown meaningful shifts from sedentary behaviour towards light PA and MVPA [16, 20]. In this regard, some PAL lessons have achieved levels of MVPA comparable to those observed during physical education classes [22].

Evidence on the effectiveness of PAL in the context of secondary education is more limited and less conclusive than in primary education. Regarding overall PA, most studies evaluated multi-component interventions that incorporate PAL in conjunction with other components such as active breaks or active recess [23, 24]. Despite including PA doses beyond PAL, these studies did not observe significant increase in overall PA following the implementation of their interventions. However, some studies report positive effects. Nevertheless, some studies report modest or context-specific effects. For example, Kolle et al. [23] observed that, although both the intervention and control groups increased sedentary time and reduced average PA levels, the decline was smaller in the intervention group, with statistically significant differences between groups. In contrast, Bergen et al. [25], who implemented PAL as a stand-alone strategy, reported a small increase in PA. However, this finding was based on subjective self-reported measures, such as self-perception of PA. With respect PA during school hours, the available evidence suggests a generally positive trend following interventions that include PAL, particularly regarding average school-time PA and MVPA [23, 24]. Findings remain inconsistent, as other studies, such as Gammon et al. [26], reported no significant changes in school PA levels after PAL implementation. Similar discrepancies have been reported when analysing PA levels during PAL sessions themselves. While Gammon et al. [26] found no significant compared to baseline levels, the study by Ruiz-Hermosa et al. [27] showed a reduction in sedentary time and significant increases in overall PA and MVPA on PAL days compared to non-PAL days.

Despite the growing interest in PAL as a strategy to increase PA levels in the educational context, the available evidence in secondary education remains characterised by heterogeneity and significant methodological gaps, particularly about PA levels during the school day. Specifically, many studies rely on subjective or aggregated measures of PA, which limits the ability to capture more subtle or context-specific effects. Furthermore, few studies have examined intra-individual variability by comparing PA levels on days with and without PAL implementation, making it difficult to isolate the acute contribution of PAL lessons across different time frames. In this context, the present study provides novel evidence by specifically analysing the impact of a PAL lesson in secondary education, using accelerometer-based measures of PA, and a design that allows us to compare students’ responses in different time contexts. The present study had as main objectives: (i) To assess the effect of a PAL intervention on PA levels during the school day in secondary school students, in comparison with the control group; ii) Compare within the intervention group a day without PAL before the intervention with the same day with PAL during the intervention, and analyse the effects of a PAL lesson on PA levels throughout the full day and during school hours. In addition, as a secondary objective, we aim to describe the average distribution of PA intensities during the PAL lessons.

## Methods

### Study design and participants

**Fig. 1:**
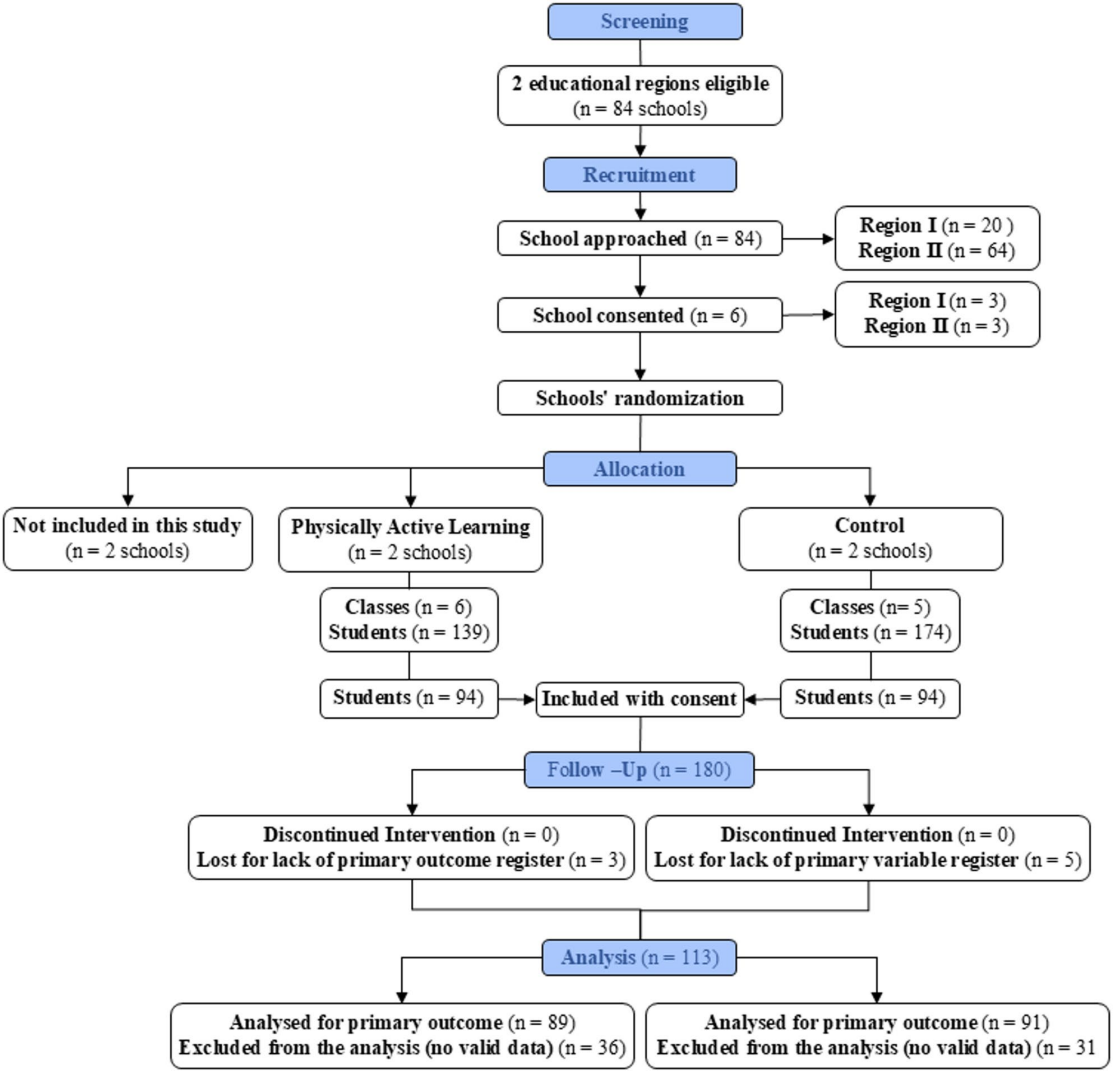
Flow diagram - CONSORT

The present study emerges from the cluster randomised controlled trial ACTIVE CLASS study [28] (registration number: NCT05891054), which followed the CONSORT statement for cluster randomized trials [Additional file 1]. Schools were stratified by educational region prior to randomisation. In each region, three schools agreed to participate and were randomly assigned to one of the three arms of the ACTIVE CLASS study, resulting in one school per group within each region. The present manuscript uses data exclusively from the PAL and control group, resulting in a total of four schools being included. Figure 1 illustrates the flow chart of participants.

The PAL group received a PAL lesson in the subject of mathematics for 16 weeks, while the control group did not experience changes to their usual teaching methodology. PA and sedentary time were assessed in both groups prior to the intervention and in the last week of the intervention. This study protocol has been reviewed and approved by the Bioethics Committees of the Andalusian Government (Cadiz, Spain), and the Bioethics and Biosafety Committees of the University of Extremadura (Caceres, Spain).

All students who met the following inclusion criteria were invited to participate: (i) studying seventh or eighth grade (12–14 years old); (ii) having no physical disability or health problem that could limit PA levels. Parents or legal guardians were given an explanation of the study in advance and written informed consent was obtained from them. Of the 313 students who were invited to participate, 180 provided informed consent, of whom 113 students (approximately 63%) provided valid accelerometer data and were included in the main analyses: PAL group *n* = 60 (*n* = 24 boys); control group *n* = 53 (*n* = 22 boys).

To address the secondary objective of this study and analyse the distribution of sedentary time and PA intensities specifically during PAL lessons, two complementary data sources were used: (i) Accelerometer data exclusively from the PAL lesson segment collected during the intervention week, in which students wore the device continuously for seven consecutive days. (ii) Accelerometer data from a separate school day, in which students wore the device exclusively during a single PAL class. A total of 236 PAL lesson records were collected, of which 215 records with valid data were included in the analysis.

### PAL intervention

The PAL intervention aimed to integrate PA into mathematics lessons without altering the established curriculum. To achieve this, the research team met with the mathematics teacher once a week to collaboratively plan each PAL lesson. During these coordination sessions, the mathematical content to be covered was defined, and the physical activities to be incorporated into the lesson were designed together. This collaboration allowed the research team’s expertise in incorporating PA through movement, jumping, and throwing to complement the teacher’s knowledge of the curriculum. As a result, the physical activities were aligned with the learning objectives, ensuring that the lesson maintained its academic rigor.

During the 16-week intervention period, a weekly PAL lesson lasting 55–60 min was conducted outside the classroom. These lessons were held in the school playground, indoor gymnasium, or the school’s outdoor common areas. To ensure consistency, each class group always participated in the PAL lessons on the same day and at the same time throughout the 16 weeks. Mathematics teachers were responsible for delivering the lessons, but a member of the research team was present at each session to support the teacher and ensure the lessons were conducted properly.

The structure of each PAL lesson remained consistent throughout the intervention. The teacher began by introducing the content to be covered, then explained and developed each activity. Each lesson included two or three activities that incorporated movement into mathematical exercises through games and collaborative or competitive tasks. These activities required students to remain physically active while solving mathematical problems, involving continuous movement such as walking or running between stations, collecting or manipulating objects, or performing simple movement-based responses linked to academic tasks. Although lesson duration and structure were consistent, the specific activities varied across sessions and were designed to promote sustained movement during the lesson, with the intention of reducing sedentary time and eliciting at least light, and in many cases moderate-to-vigorous, physical activity. Across the intervention, PAL lessons covered key curriculum topics, including operations and problem-solving with fractions and decimals, monomials, equations, proportionality, geometry, volume measurement, and basic functions. Examples of activities that follow the methodology used in this study can be found here: https://eumoveproject.eu/physically-active-lessons-toolkit/.

### Measures

#### PA and sedentary time

Study participants were instructed to wear actigraph movement sensors (Actigraph GT3X+, Inc., Pensacola, FL, USA). The devices were attached to the non-dominant wrist and were programmed to collect accelerations at a sampling frequency of 100 Hz. Participants wore the devices for eight days, while the devices were programmed to collect activity data for seven full days. The participants were instructed to wear the device for the entire day, including sleeping. Exceptionally, they were to remove the device when it came into contact with water, for example, in the shower. Once the data collection was finished, devices were returned to the research facilities for data extraction and processing. Raw sensor data were processed using the open-source R package GGIR (version 3.2.6) [29].

Acceleration data was calibrated using the automatic algorithm by Van Hees et al. [30]. Non-wear time was detected based on the standard deviation and range of the raw accelerations following the default methods in GGIR, i.e., the updated version of the algorithm by Van Hees et al. [31] as described here: https://wadpac.github.io/GGIR/articles/chapter3_QualityAssessment.html#nonwear_approach-2023. As standard in GGIR, time gaps potentially present in the accelerometer raw data were imputed, and clipping time characterized by unusual sustained high accelerations was detected and classified as invalid. For acceleration features, Euclidean Norm Minus One *G* with negative values rounded to zero (ENMO) [32], a validated acceleration-based metric derived from raw triaxial data and commonly used to estimate PA intensity, the angle of the z-axis of the device relative to the horizontal plane, and step counts were derived from the raw accelerations.

PA intensity was classified using previously calibrated and validated cut-points: 35 mg for light intensity, 200 mg for moderate intensity, and 700 mg for vigorous intensity [33, 34], originally developed in children and adults and subsequently recommended for young people [35]. A school day segment or PAL segment was considered valid if at least 90% of the time in that segment was classified as awake, and the device was worn for at least two-thirds of the segment duration. A full day was considered valid if the device was worn for at least 10 h during the time classified as awake. The detailed methods used in the R package GGIR are fully specified in the configuration parameters table, which can be found in the Supplement file.

The following criteria were established to determine whether a participant had valid data to be included in the analyses: (i) Present at least three valid school day segments at both time points in the case of the analysis of the effect of the intervention on the school day. (ii) Present a valid full day corresponding to the day of PAL lesson delivery at both time points for the analysis of the effect of a PAL lesson on full-day PA. (iii) Present a valid school day segment on the specific day of PAL delivery at both time points in the analysis of the effect of a PAL lesson on the school day. No missing data imputation method was implemented.

### Statistical analysis

Preliminarily, to examine potential selective dropout, independent-sample t-test was conducted at baseline comparing participants included in the final analyses with those excluded due to invalid accelerometer data. No statistically significant differences were observed in sedentary time, light PA, or MVPA.

As students were nested within classes, intervention effects were analysed using linear mixed-effects models. The data were treated as a hierarchical structure in which repeated observations (pre and post) were nested within students, and students were nested within classes. For the first objective (Group × Time design), a linear mixed-effects model was fitted for each dependent variable. The model included four fixed effects (intercept, group, time, and group × time interaction), with sex, age, and city included as covariates. A random intercept was specified at the class level to account for between-class variability. A diagonal covariance structure was applied for the repeated measures at the individual level, and degrees of freedom were estimated using the Satterthwaite approximation. For clarity, the Results section primarily reports the Group × Time interaction effect, which represents the intervention effect (i.e., differential change between experimental and control groups), together with the variance component of the random intercept reflecting between-class variability.

Regarding the second objective, only participants from the experimental group were included, reducing the number of level-2 units to six classes. Given the limited number of clusters, estimation of random intercept variance proved unstable. Therefore, a linear mixed-effects model including Time as a fixed factor and sex, age, and city as covariates was implemented, specifying the repeated structure at the individual level while statistically accounting for data dependency without estimating between-class random variance components.

Effect size was assessed by partial eta squared (η^2^), with reference values of 0.01 (small effect), 0.06 (medium effect) and 0.14 (large effect) [36]. The significance level was set at *p* < 0.05 in all three analyses. To address the secondary objective of the study, a descriptive analysis was conducted to present the mean distribution of PA intensities during PAL lessons. For this purpose, the mean percentage of time spent at each PA intensity was calculated from two PAL lessons per participant. All procedures were carried out using the Statistical Package for Social Sciences (SPSS) version 26 for Windows (IBM, Armonk, NY, USA).

## Results

### Effect of the PAL intervention on weekly PA during the school day

Table 1 shows the effect of the PAL intervention on different weekly average levels of PA during school hours. Since students came from four schools with varying school day lengths (ranging from 360 to 390 min), the data are presented in both minutes per week and as a percentage of total school time to facilitate comparison between schools. In both cases, the distribution of PA intensities followed the same general trend. The results indicate non-significant trends towards a slight decrease in sedentary time and an increase in MVPA in both groups. Light PA showed a non-significant increase in the control group, while remaining relatively stable in the experimental group. None of these changes reached statistical significance in the group-time interaction for any of the analysed PA intensities.

**Table 1:** Differences in estimated weekly school-time PA intensities (daily averages in minutes and percentages) between control and experimental groups

Variable	Control group (*n* = 60)		Experimental group (*n* = 53)		Interaction group x time			Between-Class Intercept Variance	
	Pre -Intervention	Intervention	Pre - Intervention	Intervention	*p*	η^²^	F	β	*p*
**School-Time PA (minutes/day)**									
Sedentary time	252.0 ± 4.9	243.3 ± 6.0	254.2 ± 5.0	251.7 ± 6.3	0.483	0.003	0.494	68.396	0.199
Light PA	110.4 ± 4.1	116.4 ± 4.9	110.7 ± 4.2	110.1 ± 5.1	0.350	0.005	0.877	49.322	0.211
MVPA	14.7 ± 1.4	17.2 ± 1.7	12.0 ± 1.5	15.1 ± 1.8	0.794	0.000	0.069	7.261	0.202
**% of School-Time in PA intensities**									
Sedentary time	66.8 ± 1.3	64.6 ± 1.6	67.5 ± 1.3	66.8 ± 1.7	0.521	0.002	0.414	4.659	0.207
Light PA	29.3 ± 1.1	30.9 ± 1.3	29.3 ± 1.1	29.2 ± 1.4	0.371	0.004	0.803	3.446	0.215
MVPA	3.9 ± 0.4	4.6 ± 0.5	3.2 ± 0.4	4.0 ± 0.5	0.748	0.001	0.103	0.529	0.202

*PA *Physical activity, *MVPA *Moderate-to-vigorous physical activity. η² = Partial eta squared. Data are presented as mean (± standard deviation) or percentage, as stated. *P*-value of the interaction group*time indicates differences in the changes over time of the experimental group compared to the control group. *P*-values of between-class variability indicate differences in the dependent variable between classes

### Effect of a PAL lesson on PA levels during a full day and school hours

In relation to the second objective of the present study, Table 2 shows the effect of a PAL class on daily PA levels and during school hours, comparing the same day with and without the PAL class, exclusively in the experimental group. Since the participants’ wear time was longer on the day they implemented PAL compared to the day without PAL (951.3 min and 935.9 min respectively), the results for the full day are expressed in daily minutes and as a percentage relative to wear time.

**Table 2:** Differences in PA intensities between a day without a PAL lesson and the same day with a PAL lesson during full-day and school-hours in the experimental group

	Day without PAL lesson	Day with PAL lesson	Time Effect		
	**Full-Day (** ***n*** ** = 47)**		*p*	η^2^	F
**min/day**					
Sedentary time	645.79 ± 15.02	655.93 ± 15.02	0.634	0.002	0.228
Light PA	259.12 ± 9.03	261.30 ± 9.03	0.865	0.000	0.029
MVPA	31.01 ± 3.34	34.06 ± 3.34	0.520	0.004	0.416
**% wear-time**					
Sedentary time	69.02 ± 1.15	68.78 ± 1.15	0.876	0.000	0.024
Light PA	27.71 ± 0.91	27.59 ± 0.91	0.930	0.000	0.008
MVPA	3.27 ± 0.36	3.64 ± 0.36	0.469	0.005	0.528
	**School-Time (** ***n*** ** = 50)**				
**min/day**					
Sedentary time	255.14 ± 5.60	245.57 ± 5.60	0.229	0.014	1.462
Light PA	108.16 ± 4.52	112.09 ± 4.52	0.540	0.004	0.378
MVPA	2.37 ± 0.94	14.56 ± 0.94	< 0.001	0.444	84.57
**% of school-time**					
Sedentary time	68.53 ± 1.41	65.93 ± 1.41	0.196	0.016	1.696
Light PA	29.11 ± 1.21	30.13 ± 1.21	0.551	0.003	0.357
MVPA	0.64 ± 0.26	3.94 ± 0.26	< 0.001	0.441	83.741

*PA *Physical activity, *PAL *Physically active learning, *MVPA *Moderate-to-vigorous physical activity. η² = Partial eta squared. Data are presented as mean (± standard deviation) or percentage, as stated. The *p*-value and *η*² refer to the effect of time, comparing physical activity levels between the day with PAL and the day without PAL within the intervention group

The results for the full day, expressed as an average daily total in minutes, show small, non-significant increases in sedentary time, light PA, and MVPA on the day the PAL lesson was implemented, compared to the day without PAL lesson. Similarly, when expressed as a percentage of wear time, the data show non-significant trends a slight decrease in sedentary time and light PA, as well as a non-significant increase in MVPA when PAL lesson was applied.

When focusing specifically on school hours, the data show the same trend when expressed as an average in minutes and as a percentage relative to the school time. Non-significant reduction in sedentary time and non-significant increases in light PA was observed, along with statistically significant increases in MVPA (*p* < 0.001, η² = 0.444 and *p* < 0.001 η² =0.441 respectively, both corresponding to large effect sizes) on the day the PAL lesson was implemented, compared to the day without the PAL class.

### Average distribution of PA intensities during PAL lessons

**Fig. 2:**
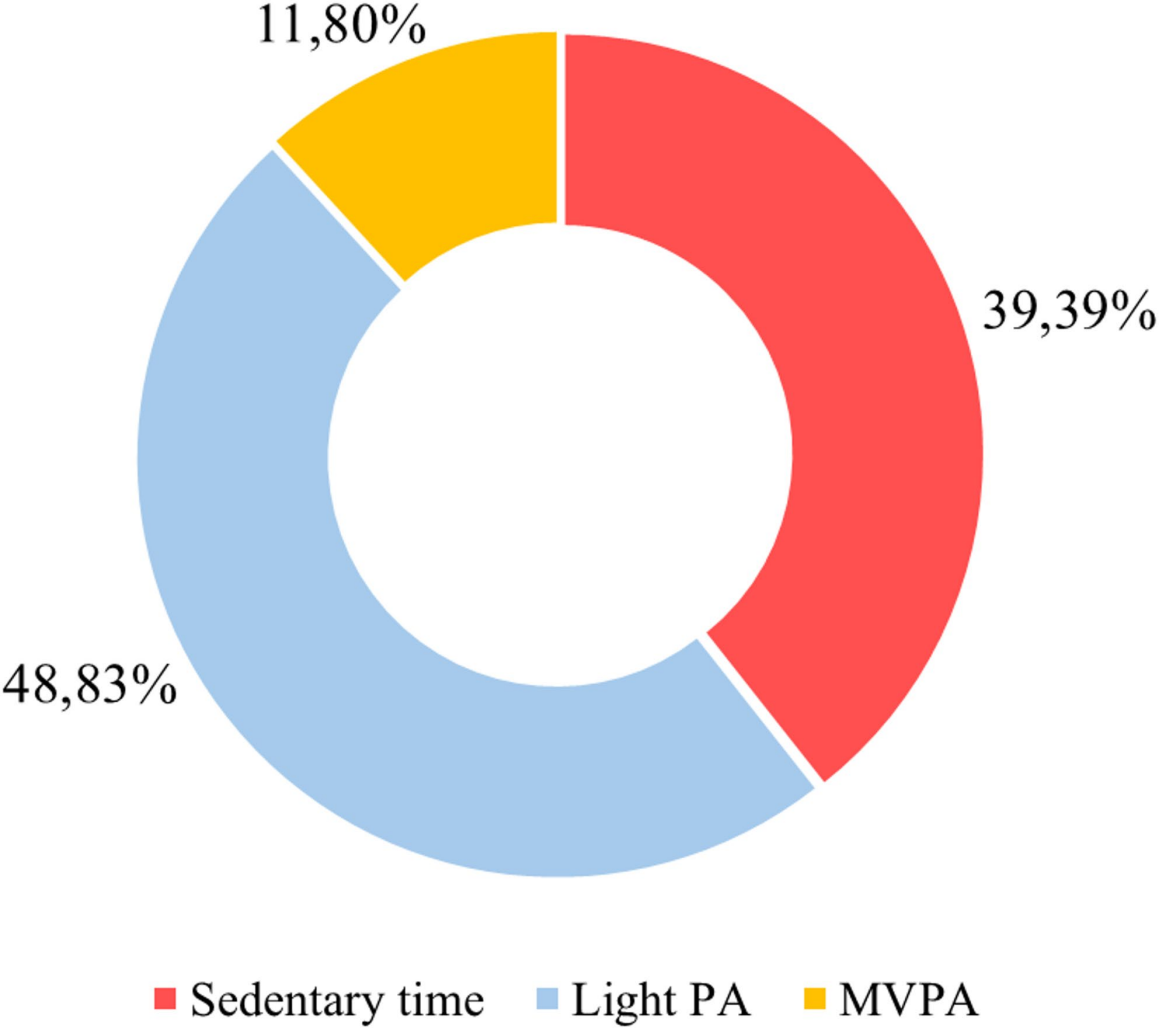
Average distribution of different PA intensities during PAL lessons

As a secondary objective, we aimed to analyse the distribution of sedentary time and PA intensities specifically within the PAL lessons. Figure 2 presents the average distribution of different PA intensities during PAL lessons, where the predominant behaviour was light PA, representing 48.83% of the total lesson time. Sedentary time accounted for 39.39% of the total time, while 11.8% of the remaining time was spent on MVPA.

## Discussion

The present study examined the impact of a PAL-based intervention on PA levels in secondary school students. In particular, the effects of the intervention on PA levels during the school day were evaluated, as well as the specific effect of a PAL lesson on PA levels in a full day and, more specifically, during school hours. As a secondary objective, the mean distribution of time students spent on different PA intensities during two PAL lessons was described. The results showed no statistically significant differences in PA levels during the school day after the intervention, nor in daily PA levels when comparing a day with and without a PAL lesson. However, there was a significant effect of the implementation of a PAL lesson on PA levels during the school day, reflected in a significant increase in time spent in MVPA.

With respect to the first objective of this research, the results suggest a slight non-significant reduction in sedentary time and non-significant increases in MVPA in the experimental group after the implementation of PAL compared to the control group. These results contrast with those reported in previous studies, which generally found a positive effect of PAL on increasing students’ PA levels during school hours in both primary [18, 20, 21, 37] and secondary [23, 24] education. However, it is important to consider several methodological differences that might help to explain this lack of agreement with our results. In the case of research carried out in primary education, most studies do not use objective measures of PA, instead opting for less precise instruments such as pedometers or heart rate monitors. These instruments, while useful for estimating total movement volume, do not allow for accurate discrimination between different PA intensities, which compromises both the validity and sensitivity of the results. In this regard, the systematic review by Daly-Smith et al. [18] identified only two studies that assessed PA using accelerometry, while the review by Norris et al. [37] reported only one such study. These limitations should be considered when comparing the effects of PAL interventions, as the type of instrument used to measure PA could influence the precision of the estimates and the ability to detect significant changes. In secondary education, although the available evidence uses accelerometry as a measure of PA, there are other methodological variables, such as the design of the intervention, that must be considered when interpreting the results. For example, the research by Kolle et al. [23] and Schmidt et al. [24], conducted research using multicomponent interventions, where in addition to PAL lessons, they include other PA doses in their intervention, such as active breaks, reaching a total of 120 min per week of intervention during periods of seven to nine months. In contrast, research such as that by Gammon et al. [26], which was based exclusively on PAL lessons implemented autonomously by teachers after a training period, did not observe significant changes in school PA levels, despite an average of one 60-minute lesson per day for six weeks. Taken together, these findings suggest that differences in intervention dose may partly explain the heterogeneous results observed across studies, and that the 60 min of PAL per week accumulated over 16 weeks in the present intervention may represent one of several factors influencing changes in students’ physical activity levels [16]. In this sense, student and teacher characteristics, as well as other school-contextual factors operating across the school week also likely to play a role. Among these, previous research on PAL has highlighted aspects related to teachers’ experience with PAL, their confidence in conducting movement-based activities, and the degree of integration of such approaches into the broader school context as relevant sources of variability [38].

Beyond intervention design, the present study contributes to methodological advances in PAL research by using accelerometer-measured to examine PA at different temporal levels. While weekly average analyses were used to assess overall changes in school-based PA, the inclusion of a within-day approach allows a more fine-grained examination of the acute effects of PAL lessons on the specific day of their implementation. Building on this approach, this study evaluated PA levels both in the full day and in school hours, exclusively in the experimental group. The results showed no significant difference in overall daily PA between days with and without PAL. Although the results showed a trend towards a reduction in sedentary time and an increase in MVPA when the PAL lesson was implemented, these variations did not reach statistical significance. To the authors’ knowledge, there are no previous studies that have compared this effect by examining at a single day with PAL versus a day without intervention. However, the present findings can be contextualised from research such as Kolle et al. [23] and Schmidt et al. [24], who analysed daily PA levels averaged over at least two days during the baseline period and the intervention period. In both cases, despite the interventions being multicomponent with a higher dose of PAL than in our study, no significant improvements in overall PA were observed either. In fact, both investigations reported an increase in sedentary time. Kolle et al. [23] further reported a decrease in both light PA and MVPA, while Schmidt et al. [24] observed an overall increase in overall PA. These results suggest that a single PAL lesson may have a limited impact on full-day PA, as PA levels outside school hours are likely shaped by other contextual factors, such as after-school activities or the family and social environment [39]. Additionally, it is possible that the absence of significant differences in full-day MVPA may partly reflect a compensatory pattern, whereby increases in activity during school hours are offset by reductions later in the day, as proposed by the ActivityStat hypothesis and reported in some school-based PA interventions [40, 41]. Future research should further explore activity patterns beyond school hours to better understand how school-based PA interventions interact with students’ overall daily behaviour.

Importantly, this study extends previous PAL research by adopting a within-day analytical approach, allowing the examination of the acute effects of a single PAL lesson on PA during the same school day. This temporal resolution provides novel insight into when and how PAL influences students’ activity patterns, beyond averaged weekly or intervention-period estimates commonly reported in the literature. Accordingly, when analysing the effect of the PAL lesson on the day it was implemented during school hours, the results of the present study show non-significant reductions in sedentary time, and non-significant increases in light PA, together with a significant increase in MVPA. Although few studies have specifically addressed this type of analysis, the research by Norris et al. [19] offers a point of comparison by assessing the impact of a PAL lesson on school PA on the same day of implementation, comparing an experimental group with a control group. In their study, however, no significant differences were observed between groups in terms of PA levels during the school day. This discrepancy with our findings could be partly related to differences in intervention characteristics, including intervention dose, as Norris et al. [19] applied a 30-minute lesson, whereas our intervention was based on 60-minute lessons. In this line, the pilot study by Ruiz-Hermosa et al. [27], which applied an identical intervention to that in our research, offers complementary results. Although they did not analyse the day of implementation specifically, they compared the mean levels of school PA of the experimental group on at least three days during the week prior to the intervention with those during the final week of the programme, when a PAL was implemented. They observed a significant decrease in sedentary time, as well as increases in light PA and MVPA. Together with the findings of the present study, these results suggest that a 60-minute weekly PAL lesson may have the potential to significantly improve PA levels during school hours on the day it is implemented. However, whether such effects extend to the full day is likely to depend on additional factors, such as the overall intervention dose and the contextual and socio-economic characteristics of the students.

From a practical perspective, these findings indicate that PAL may represent a useful pedagogical resource to be integrated within academic lessons, providing greater variety in teaching approaches while potentially contributing to increases in MVPA during school hours. Thus, the inclusion of PAL within the school timetable may help support schools in moving towards current PA recommendations for children and adolescents during the school day, within the constraints of the educational context.

As a secondary objective, this study analysed the distribution of PA intensities during PAL lessons. The results showed that light PA was predominated, accounting for 48.83% of the lesson time, followed by sedentary behaviour (39.39%) and MVPA (11.8%). These findings are consistent with those of Ruiz-Hermosa et al. [27] and Seljebotn et al. [22], who also found a higher proportion of light PA during PALs (40% and 38%, respectively). Furthermore, both studies compared PAL lessons with physical education classes, and found no significant differences in the intensity distribution, suggesting that PALs can provide an additional hour of PA without compromising academic content. On the other hand, research such as Gammon et al. [26] and Johansen et al. [42] reported that sedentary behaviour was predominant during PAL lessons (70% and 55.6%, respectively), which contrasts with our results. This disparity could be due to the degree of teacher autonomy in implementing PALs. In the studies by Ruiz-Hermosa et al. [27] and Seljebotn et al. [22], teachers received continuous support from the research team. In contrasts, in the studies by Gammon et al. [26] and Johansen et al. [42], the lessons were implemented autonomously after a period of training. However, the intensity of the training also seems to play a role. Thus, Johansen et al. [42] observed a 20.7% MVPA time after a one-year training programme, compared to the 1.6% reported by Gammon et al. [26] after only two training sessions. These findings suggest that providing ongoing support to teachers and investing in higher-quality training may be important strategies for enhancing the active component of PAL classes and optimising the time devoted to PA within academic settings.

### Strengths and limitations

This study has several notable strengths. Firstly, it was conducted as a cluster randomised controlled trial, a design rarely used in PAL research and particularly challenging to implement in school settings, especially when introducing an innovative pedagogical approach such as PAL. Furthermore, it was implemented in secondary education, an area where evidence on the impact of PAL is limited and inconclusive. Additionally, the use of accelerometry to assess PA levels represents an advantage over other studies that have used indirect methods, such as pedometers or heart rate monitors, which are less sensitive and unable to discriminate between intensities as well. Finally, this study includes intra-individual analyses comparing the PA levels of the same students on days with and without a PAL lesson. This provides more precise and novel evidence of the acute effects of these types of intervention in real school contexts.

However, this study has some limitations that should be considered. Firstly, although accelerometry was used as an assessment tool, implementing it with an adolescent population presented certain logistical challenges, such as some participants not adhering to the continued use of the device. This reduced the final sample size, which, while sufficient for the analyses performed, may have reduced the statistical power to detect significant effects. Secondly, the intervention may not have been sufficiently intensive to induce sustained changes in PA levels, particularly compared to previous longer-term, more frequent or more comprehensive interventions. In addition, although key structural aspects of the intervention were supervised (i.e., lesson duration, frequency, and support from the research team during each session), implementation fidelity was not formally assessed using standardized observation tools or adherence checklists, nor was the effective intensity of the lessons systematically monitored. Therefore, variability in how PAL activities were delivered and implemented across sessions cannot be ruled out, which may have influenced the consistency of the observed effects. Finally, the study was conducted with a single group of participants in schools located in only two Spanish provinces, which may limit the generalisability of the results to other curricular, territorial or educational contexts. Moreover, as participation was voluntary and only a small proportion of the schools invited agreed to take part, it is possible that the sample included schools more predisposed to implementing innovative pedagogical approaches, which may also limit the external validity of the findings.

## Conclusion

PAL may contribute to increasing PA during the school day at specific times, although its effects do not appear to extend to the rest of the day or accumulate throughout the week, possibly due to the influence of methodological, contextual, or personal factors.

From a practical perspective, this study provides evidence of the feasibility of integrating PAL strategies into core subjects such as mathematics in secondary education, particularly when there is active collaboration between teachers and the research team. Future research should explore interventions with greater frequency or duration, expand the sample size to facilitate the generalisation of results, develop strategies to improve adherence to accelerometer use, and further examine how school-based PAL interventions interact with students’ overall daily activity patterns across different time domains. Additionally, examining how aspects related to the quality of implementation and the way PAL activities are delivered within lessons may influence students’ engagement and PA responses could help refine and optimise future interventions. Despite its limitations, this study provides valuable insights into the immediate effects of PAL in real secondary education settings, reinforcing its potential as a school strategy to reduce sedentary behaviour and promote PA without compromising academic learning.

## Supplementary Information

Supplementary Material 1.

## Data Availability

The datasets used and/or analysed during the current study are available from the corresponding author on reasonable request.

## Abbreviations

PAL: Physically Active Learning
PA: Physical Activity
MVPA: Moderate-Vigorous Physical Activity
